# Self‐Tuning n‐Type Bi_2_(Te,Se)_3_/SiC Thermoelectric Nanocomposites to Realize High Performances up to 300 °C

**DOI:** 10.1002/advs.201700259

**Published:** 2017-08-11

**Authors:** Yu Pan, Umut Aydemir, Fu‐Hua Sun, Chao‐Feng Wu, Thomas C. Chasapis, G. Jeffrey Snyder, Jing‐Feng Li

**Affiliations:** ^1^ State Key Laboratory of New Ceramics and Fine Processing School of Materials Science and Engineering Tsinghua University Beijing 100084 P. R. China; ^2^ Department of Materials Science and Engineering Northwestern University Evanston IL 60208 USA

**Keywords:** bismuth‐telluride‐selenide, device figure of merit, self‐tuning, thermoelectrics

## Abstract

Bi_2_Te_3_ thermoelectric materials are utilized for refrigeration for decades, while their application of energy harvesting requires stable thermoelectric and mechanical performances at elevated temperatures. This work reveals that a steady *zT* of ≈0.85 at 200 to 300 °C can be achieved by doping small amounts of copper iodide (CuI) in Bi_2_Te_2.2_Se_0.8_–silicon carbide (SiC) composites, where SiC nanodispersion enhances the flexural strength. It is found that CuI plays two important roles with atomic Cu/I dopants and CuI precipitates. The Cu/I dopants show a self‐tuning behavior due to increasing solubility with increasing temperatures. The increased doping concentration increases electrical conductivity at high temperatures and effectively suppresses the intrinsic excitation. In addition, a large reduction of lattice thermal conductivity is achieved due to the “in situ” CuI nanoprecipitates acting as phonon‐scattering centers. Over 60% reduction of bipolar thermal conductivity is achieved, raising the maximum useful temperature of Bi_2_Te_3_ for substantially higher efficiency. For module applications, the reported materials are suitable for segmentation with a conventional ingot. This leads to high device *ZT* values of ≈0.9–1.0 and high efficiency up to 9.2% from 300 to 573 K, which can be of great significance for power generation from waste heat.

## 1 Introduction

Thermoelectric materials that enable direct exchange between heat and electrical energy have drawn increasing attention not only for scientific interest but also for industrial importance.^1^, ^2^, ^3^, ^4^ The efficiency of a thermoelectric material is characterized by dimensionless figure of merit *zT*, defined as *zT* = α^2^ *σT*/κ, where α, σ, *κ*, and *T* are the Seebeck coefficient, electrical conductivity, thermal conductivity, and absolute temperature, respectively.^5^, ^6^ In the past two decades, many advanced thermoelectric materials have been developed thanks to the unceasing efforts to *zT* enhancement, especially the maximum values of *zT* (*zT*_max_).^7^, ^8^, ^9^, ^10^ However, achieving high device *ZT* values in a large temperature range is more important from a view point of increasing device energy conversion efficiency.^11^ Different from material *zT*, device *ZT* is approximately the temperature average of the material *zT* but also includes the effect of thermoelectric compatibility.^12^

As the state‐of‐the‐art thermoelectric material, Bi_2_Te_3_‐based ingots show high *zT* at room temperature (RT) and have been employed in industry for decades in solid‐state cooling.^13^ Recently, the applications of Bi_2_Te_3_‐based thermoelectric materials in waste‐heat recovery in the low‐mid temperature range are receiving more and more attention.^14^ With two‐thirds of the primary energy is lost as waste heat (low‐temperature region at 100–300 °C constitutes over 50%),^3^ there is a high demand for stable thermoelectric performance in low‐temperature range up to 300 °C. Although some new thermoelectric materials have been developed with promising performance in the temperature range of 200 to 300 °C so far,^15^, ^16^, ^17^ optimizing the thermoelectric performance of the widely used Bi_2_Te_3_‐based alloys in this temperature range is technically attractive. Presently, shifting the *zT* to higher temperatures is still challenging considering the small band gap of Bi_2_Te_3_ (*E*_g_ = 0.13 eV), in which intrinsic excitation of minority carriers happens easily at high temperatures and thus leads to a rapid decrease of Seebeck coefficient and huge increase of the thermal conductivity.^18^, ^19^ Increasing the carrier concentration is one way to suppress the bipolar effect, but unfortunately would also lower the *zT* at low temperatures as the corresponding optimal carrier concentration of *zT*_max_ increases with temperature. One solution in dealing with that is segmenting functionally graded materials^20^ or self‐tuning using a dopant that has temperature‐dependent solubility.^21^ Herein, we show that by combining self‐tuning carrier concentration and segmentation design, high device *ZT* and conversion efficiency in the whole temperature range from RT to 300 °C in Bi_2_Te_2.2_Se_0.8_ could be achieved.

In this work, Cu/I was adopted as the temperature‐dependent dopants to self‐tune the carrier concentration. The temperature‐dependent carrier concentration results in an increase of the electrical conductivity, a smaller decrease of the absolute values of Seebeck coefficient, and a 60% reduction in bipolar thermal conductivity at high temperatures, which are all advantageous for the *zT* enhancement at elevated temperatures. Moreover, the lattice thermal conductivity in the whole temperatures range is also decreased, due to the precipitation of CuI nanoparticles beyond the solubility limit. Consequently, stable thermoelectric performance of n‐type Bi_2_Te_2.2_Se_0.8_ alloys with a *zT* plateau of ≈0.85 between 200 and 300 °C was achieved. By segmenting with commercial ingots, a high device *ZT* over 0.9 and efficiency of 9.2% could be achieved in a wide temperature range from RT to 300 °C, which would be very promising for low‐temperature waste‐heat recovery applications.

## 2 Results and Discussion

### 2.1 SiC Dispersion

**Figure 1:**
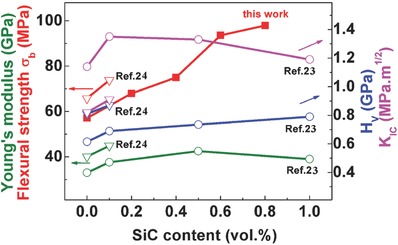
Mechanical properties of Bi_2_(Te,Se)_3_–*x* vol% SiC samples, with a summary of our previous works,^23^, ^24^ where *H*_v_ is the Vickers hardness and *K*_IC_ is the fracture toughness.

SiC nanodispersion has been widely demonstrated to be effective in enhancing the mechanical strength along with the thermoelectric performance of Bi_2_Te_3_‐based alloys.^22^, ^23^, ^24^, ^25^ Herein, SiC nanoparticles were employed to improve the mechanical stability of Bi_2_Te_2.2_Se_0.8_. No secondary phase could be identified in Bi_2_Te_2.2_Se_0.8_–*x* vol% SiC (*x* = 0, 0.2, 0.4, 0.6. 0.8, and 1.0) samples within the limits of our X‐ray diffraction (XRD) analysis (Figure S1, Supporting Information). **Figure** **1** summarizes the effects of SiC on mechanical properties of polycrystalline n‐type Bi_2_Te_3_‐based alloys in this work and previous ones.^20^, ^21^ It is clearly illustrated that addition of SiC effectively enhances the flexural strength. The flexural strength is found to increase 72% due to the addition of 1 vol% SiC particles, which could be ascribed to both the second‐phase dispersion‐strengthening effect similarly shown for the skutterudite/TiN composites^26^ and the grain refinement effect.^27^ The enhanced flexural strength is advantageous for the mechanical stability and module applications.^18^

**Figure 2:**
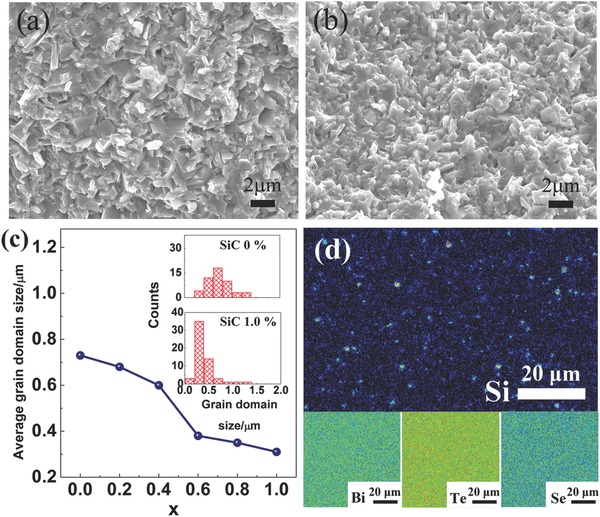
SEM images of Bi_2_Te_2.2_Se_0.8_–*x* vol% SiC fractured surfaces with a) *x* = 0 and b) *x* = 1.0, c) average “grain size” of the samples with different amounts of SiC particles (insets are the grain size distribution of the *x* = 0 and *x* = 1.0 samples), and d) EPMA mapping of Si and main elements (Bi, Te, Se) on polished surface of the *x* = 0.6 sample.

Fine grain domains (<1 µm) were observed in all the samples, and the grain domain size decreases with the amount of SiC particles. **Figure** **2**a,b shows the scanning electron microscopy (SEM) images of fractured surfaces of *x* = 0 and *x* = 1.0 samples (SEM images for other samples could be found in Figure S2 in the Supporting Information). It can be seen that the grain domain size decreases, which is also depicted in Figure 2c and Figure S3 (Supporting Information). The existence of SiC and distribution of the main elements were investigated by electronic probe microscopic analysis (EPMA) mapping on polished surfaces of the samples. Here, *x* = 0.6 sample is given as an example. As shown in Figure 2d, some Si‐rich areas which correspond to the SiC particles as well as the main elements (Bi, Te, Se) are homogenously distributed in the matrix. Moreover, the Si‐rich areas increase with the amounts of SiC addition (Figure S4, Supporting Information). The uniformly distributed SiC particles are considered to be effective for suppressing the grain growth of Bi_2_Te_2.2_Se_0.8_ matrix.

**Figure 3:**
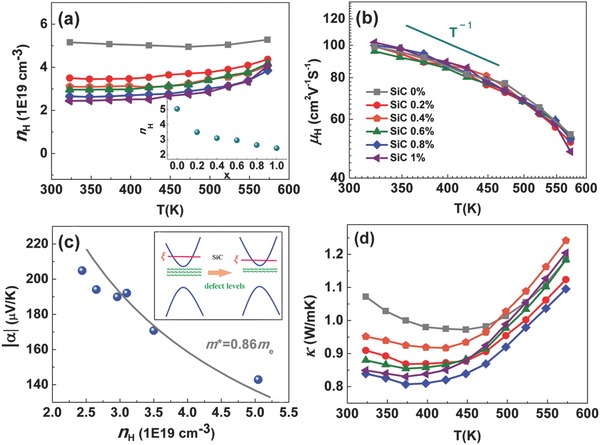
Temperature dependence of a) carrier concentration (inset shows SiC amount dependence of carrier concentration) and b) carrier mobility. c) Carrier concentration dependence of absolute Seebeck coefficient values (Pisarenko plot). d) Total thermal conductivity of Bi_2_Te_2.2_Se_0.8_–*x* vol% SiC composites.

In this system, SiC dispersion decreases the carrier concentration due to grain refinement. No interaction with Bi_2_Te_3_ structure is expected as the lattice parameters show little change upon SiC addition as shown in Table S1 in the Supporting Information. Undoped materials show a decrease in n‐type carrier concentration, suggesting SiC somehow alters intrinsic defect concentrations possibly at the interfaces of the grains (**Figure** **3**a). The carrier concentration of polycrystalline Bi_2_Te_3−_ *_x_*Se*_x_* is usually affected by the concentration of anion vacancies, antisite defects, and their interaction (donor‐like effect), which is shown in the following equation^28^, ^29^, ^30^

(1) $$2V_{\mathrm{Bi}}^{\prime \prime \prime }+3V_{\mathrm{Te}}^{¨}(V_{\mathrm{Se}}^{¨})+Bi_{\mathrm{Te}}^{\prime }(Bi_{\mathrm{Se}}^{\prime })\;\;=\;\;V_{\mathrm{Bi}}^{\prime \prime \prime }+Bi_{\mathrm{Bi}}^{*}+4V_{\mathrm{Te}}^{¨}(V_{\mathrm{Se}}^{¨})+6e^{\prime }$$

As aforementioned, SiC dispersion refines the grain size and introduces more grain boundaries. Hence, the Te dangling bonds acting as anion vacancies $\left(V_{\mathrm{Te}}^{¨}\right)$ at gain boundaries are increased, which leads to a suppression of the donor‐like effect and thus decreases the carrier concentration. However, SiC dispersion hardly changes the carrier mobility and the effective mass, as shown in Figure 3b,c. The inset in Figure 3c schematically illustrates that the change of carrier is caused by modifying the defect states. Moreover, the total thermal conductivity is decreased due to SiC addition, as shown in Figure 3d. The remained mobility and decreased thermal conductivity is advantageous for better thermoelectric performance. Detailed transport properties are given in Figure S5 in the Supporting Information.

### 2.2 *zT* Enhancement at 200–300 °C by CuI Addition

Halogens like I and Br,^31^, ^32^, ^33^ which presumably replace Te as well as metals that form interstitial defects, e.g., Cu^34^, ^35^ and Ag,^36^ have been generally adopted as donor dopants to increase the electron concentration of Bi_2_Te_3−_ *_x_*Se*_x_*. Herein, CuI was employed as the dopant source of the two individual elements, and Bi_2_Te_2.2_Se_0.8_–0.6 vol% SiC (*x* = 0.6) was chosen as the matrix's composition. Based on XRD analysis, no secondary phase was detected for the doped samples before the transport measurements (Figure S6, Supporting Information).

**Figure 4:**
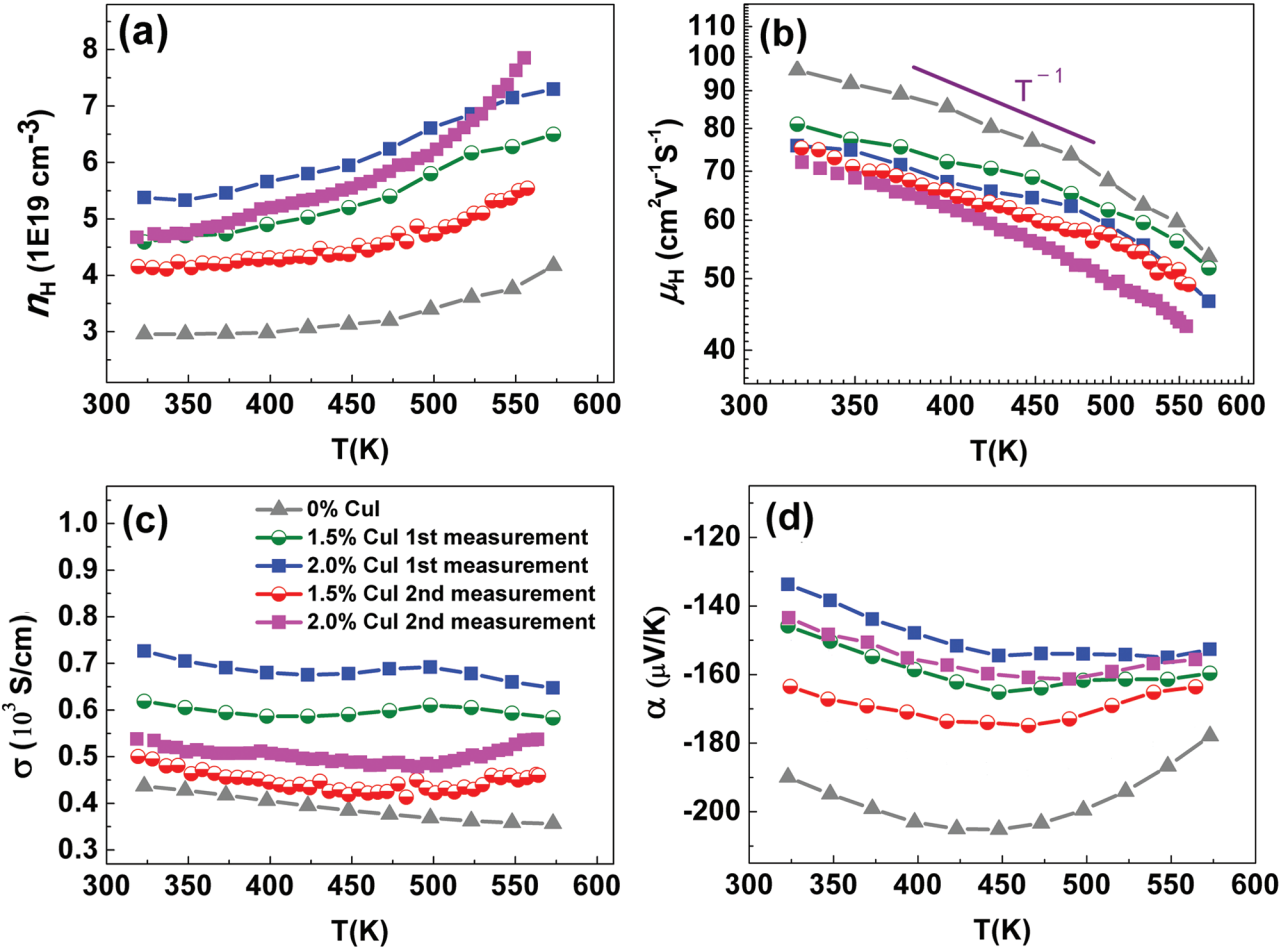
Temperature dependence of electrical transport properties of doped samples: a) carrier concentration, b) carrier mobility, c) electrical conductivity, and d) Seebeck coefficient.

Cu/I doping increased the carrier concentration and thus changed the electrical transport properties. As shown in **Figure** **4**a, the carrier concentration increases with the amount of CuI. However, the doping efficiency is relatively low (Figure S7, Supporting Information), indicating that most of the CuI do not go into the crystal structure acting as dopants but embedded in the matrix as unreacted CuI. Figure 4b shows that the mobility decreases with increasing CuI contents. Nevertheless, all the samples obey a *T* ^−1^ dependence, implying that phonon‐scattering mechanism is still dominated. Because of the increased carrier concentration, the electrical conductivity increases and the absolute values of Seebeck coefficient decrease for the doped samples, as displayed in Figure 4c,d, respectively. The increased electrical conductivity along with only a small decrease of thermal power at ≈473–573 K is of great significance for the *zT* enhancement at elevated temperatures.

The self‐tuning behavior of the increased carrier concentration with temperature for the doped samples is due to the temperature‐dependent solubility of dopants Cu/I. Pei et al. reported that Ag works well as a fast diffusing interstitial dopant in self‐tuning PbTe.^21^ Herein, the increasing trend of carrier concentration with temperature for the doped samples (*n*_H_ > 4 × 10^19^ cm^−3^) could also be explained by increasing solubility of Cu/I in the matrix. As shown in Figure 4a, the increasing trend of carrier concentration of the doped samples is more obvious than the undoped samples, which rules out the reason of minority carrier excitation below 450 K. Moreover, the self‐tuning behavior is still preserved in the second measurement, even small amounts of Cu/I dopants have precipitated out as CuI. This is because that most of the CuI precipitates are not from the dopants but come from unreacted CuI, which will be discussed later. The temperature‐induced gradient in doping concentration is advantageous for the optimal thermoelectric performance as the optimum carrier concentration typically increases with temperature.^21^

In principle, no hysteresis is expected in the transport measurement unless a compositional, structural, or microstructural change is induced on the sample during the heating/cooling cycles. In this work, we found that the second measurement gives slightly different transport properties compared with the first measurement, as shown in Figure 4a–d. Nevertheless, it should be noted that the properties become identical during the subsequent measurements (Figure S8, Supporting Information), indicating that the first measurement serves as a heat treatment process. As no hysteresis was observed in the undoped sample during the heating and cooling cycle (Figure S9a, Supporting Information), the difference between first and second measurements should be caused by the CuI.

**Figure 5:**
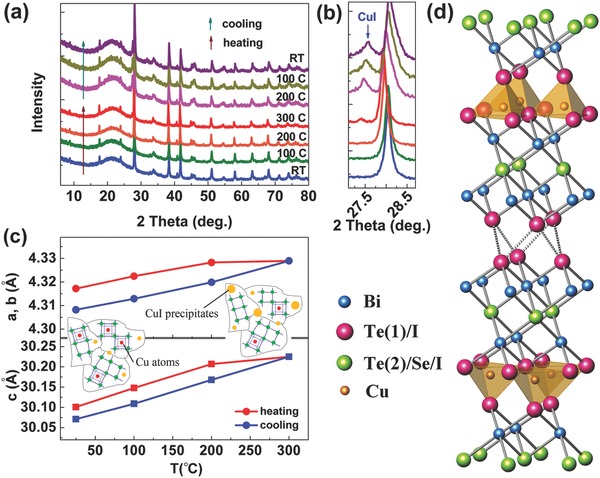
a,b) High‐temperature XRD patterns, c) lattice parameters of the CuI‐1.5 mol% sample, insets are schematice illustration of the precipitation behavior of CuI from room temperature to 300 °C, and d) schematic crystal structure of Cu and I‐doped Bi_2_Te_2.2_Se_0.8_, where Cu prefers to fill the tetrahedral sites between two Te(1) layers, and Se substitutes the Te(2) site.

The difference between the first and second measurements is due to the loss of dopants and CuI precipitation during the first measurement. As shown by the high‐temperature XRD (**Figure** **5**a,b), CuI peaks show up after heating in doped sample at above 200 °C. It has been reported that Cu atoms would go to the interstitial sites between two adjacent Te(1)–Te(1) layers due to large interatomic space and weak van der Waals interactions.^35^, ^37^, ^38^ Although it is also reported that Cu could go to Bi sites and decrease the electrons concentration,^39^, ^40^ interstitial sites are preferred in the present materials as the *c* lattice of the doped sample is found to be larger than the undoped one (Table S1, Supporting Information). The slight decrease of the slop of the *c* lattice parameter, as shown in Figure 5c, implies that small amount of Cu is lost above 200 °C, which is consistent with the decrease of carrier concentration in the second measurement. It should be mentioned that only small amounts of CuI precipitates come from the dopants, as demonstrated by the small decrease of carrier concentration and *c* lattice parameter. Significantly, most of the CuI precipitates are from the unreacted CuI (beyond doping efficiency). The unreacted CuI in the matrix before measurement is dispersive, very small particles, and with low crystallinity, which can be hardly detected by XRD. However, during the high‐temperature measurement as an annealing process, the unreacted CuI became larger particles (probably at the grain boundaries) with better crystallinity, which were detected by the XRD during the heating process. Now, we can also understand why the CuI‐2 mol% sample still shows an obvious self‐tuning behavior in the second measurement, while the CuI‐1.5 mol% sample presents a weaker increasing trend of carrier concentration (Figure 4a). Since the unreacted CuI precipitated out as detectable CuI compound, there are less CuI available for the self‐tuning behavior, especially for the CuI‐1.5 mol% sample. Nevertheless, the self‐tuning behavior in the following measurements happens the same as the second measurement, as it is independent of the in situ‐formed CuI particles that mostly related to the unreacted CuI rather than the Cu/I dopants. Figure 5d illustrates a schematic crystal structure of Cu/I‐doped Bi_2_Te_2.2_Se_0.8_, where Se prefers to occupy the Te(2) sites (Table S2, Supporting Information), I substitutes Te in both Te(1) and (2) sites, and Cu atoms are placed at the tetrahedral sites between two Te(1) layers. As I^1−^ and Te^2−^ have almost identical ionic radii (206 and 207 pm, respectively),^41^ a change in the lattice parameter due to iodine loss is not expected for this system. Notably, it is also demonstrated in Figure S9b (Supporting Information) that no hysteresis shows up if the measurement temperature is limited to 200 °C, which is consistent with the high‐temperature XRD results showing that CuI only precipitates above 200 °C.

**Figure 6:**
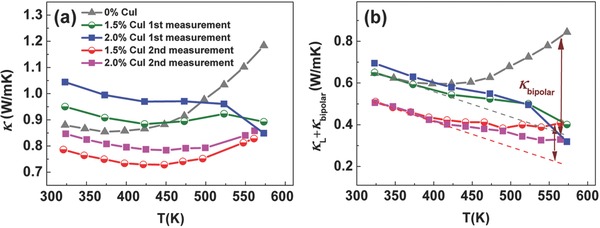
Temperature dependence of thermal transport properties: a) total thermal conductivity and b) lattice and bipolar thermal conductivity.

Cu has been widely reported to decrease the thermal conductivity of Bi_2_Te_3_‐based materials.^42^, ^43^, ^44^ More importantly, the CuI nanoprecipitates are also found to be effective in decreasing the lattice thermal conductivity in this study. As shown in **Figure** **6**a, the total thermal conductivity increases with CuI amounts at low temperatures in the first measurement. Nevertheless, the thermal conductivities are indeed decreased by CuI addition in the second measurement. It is also intriguing to find that the thermal conductivity shows a jump at 523–573 K, implying that new phonon‐scattering centers form after 523 K during the first measurement. The total thermal conductivity is composed of three parts by the equation κ = κ_e_ + κ_l_ + κ_bipolar_, in which κ_e_, κ_l_, and κ_bipolar_ are the electrical, lattice, and bipolar contributions, respectively. According to the Wiedemann–Franz law, κ_e_ = *σLT*, where σ is the electrical conductivity, *L* is the Lorenz number calculated by single parabolic band analysis,^45^ and *T* is the absolute temperature.^46^ Figure 6b shows that κ_l_ decreases for all the doped samples and keeps decreasing after the first measurement. In view of the high‐temperature results that CuI precipitates out during the first measurement, the CuI nanoprecipitates are considered as the phonon‐scattering centers. Moreover, κ_bipolar_ is extremely reduced by over 60% at 300 °C, demonstrating that the minority carrier excitation is strongly suppressed by Cu/I doping. Usually enlarging the band gap and/or increasing the carrier concentration can effectively suppress the bipolar effect. The increased carrier concentration with temperature is the reason in this work, as analysis of the diffuse reflectance data assuming direct gap transition^47^ does not indicate any major shift in optical gap by Cu/I doping (Figure S10, Supporting Information).

**Figure 7:**
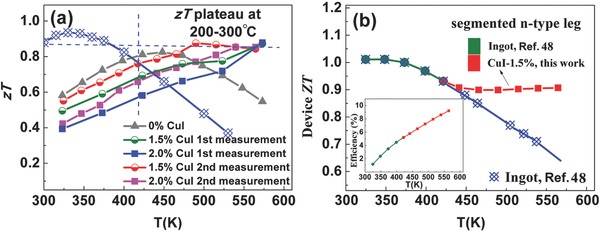
a) Temperature dependence of *zT* values, with a comparison to commercial ingots,^48^ b) comparison of device *ZT* values (with an inset of efficiency) of n‐type Bi_2_Te_2.79_Se_0.21_ ingots^48^ and that of a segmented leg composed of ingots^48^ for 300–423 K and Bi_2_Te_2.2_Se_0.8_ composite (this work) for 423–573 K. The horizontal axis (*T*) in b) is the hot side temperature with a fixed cold side temperature of 300 K.

Higher *zT* values at elevated temperatures (>200 °C) are achieved through the effective suppression of minority carrier excitation, increased electrical conductivity, and small change of Seebeck coefficient. As shown in **Figure** **7**a, the *zT* values are greatly increased from 200 to 300 °C for the Cu/I‐doped samples, showing a stable *zT* plateau of ≈0.85 at high temperatures. The maximum improvement of *zT* at 300 °C is up to ≈70%. The enhancement of *zT* values at 200–300 °C is of significance as conventional zone melting ingots usually show a *zT* peak at approximately equal RT and then decrease rapidly with temperature, and only a few materials show high *zT* in this temperature range (Figure S11a, Supporting Information).

High device *ZT* and efficiency can be achieved by combining the segments of n‐type Bi_2_Te_2.79_Se_0.21_ ingots^48^ with high *zT* at 300–423 K and the present material showing *zT* plateau at 423–573 K. As shown in Figure 7b, the calculated device *ZT* shows high values over 0.9 and retains the high values in the whole temperature range from 300 to 573 K. While without segmentation, the device *ZT* of the ingots shows a peak *ZT* at approximately equal RT and then decreases rapidly with temperature. The reason of higher device *ZT* should be due to higher material *zT* at higher temperatures of the present material and the close compatibility factors of the two segments (Figure S11b, Supporting Information). Higher efficiency could be achieved by the segmented leg than the ingots without segmentation when large temperature differences are used. By extending the use of the segmented leg to higher temperatures, the overall efficiency is increased to 9.2% (inset in Figure 7b), which rivals that of mid temperature materials (hot side temperature ≈675–900 K^49^), making Bi_2_Te_3_ competitive for waste‐heat power generation applications.

## 3 Conclusions

This work has shifted the thermoelectric performance of n‐type Bi_2_Te_2.2_Se_0.8_ to elevated temperatures up to 300 °C. Cu/I doping has effectively increased the carrier concentration with a self‐tuning behavior, which plays an important role for great suppression of intrinsic excitation along with enhancing the thermoelectric performance at elevated temperatures. Moreover, the Cu/I, beyond solubility limit, precipitates out as CuI and acts as phonon‐scattering centers, thus greatly reducing the lattice thermal conductivity in the whole temperature range. As a result, stable *zT* values around 0.85 in the temperature range of 200–300 °C are achieved for n‐type Bi_2_Te_2.2_Se_0.8_ thermoelectric material. By combining conventional ingots and the Cu/I‐doped sample together, high calculated device *ZT* values with a plateau over 0.9 and high efficiency up to 9.2% could be achieved. Meanwhile, SiC addition helps improving the flexural strength by 72%. Such a steady thermoelectric and mechanical performance would be promising for low‐temperature waste‐heat recovery applications.

## 4 Experimental Section

Bi (99.99%), Te (99.999%), Se (99.999%), SiC (≈99.99%, 100 nm), and CuI powders were mixed with a stoichiometric proportion of Bi_2_Te_2.2_Se_0.8_–*x* vol% SiC (*x* = 0, 0.2, 0.4, 0.6, 0.8, 1.0)–*y* mol% CuI (*y* = 0, 1.5, 2), and then subjected to mechanical alloying (MA) in a planetary ball mill at 450 rpm for 3 h. The mill vials were filled with high purity Ar gas (95 vol% Ar and 5 vol% H_2_). All the powder‐processing experiments were conducted in the glove box. The MA powders were then spark plasma sintered in a 12 mm diameter graphite mold at 673 K in vacuum for 5 min under an axial pressure of 50 MPa, resulting in a bulk sample with a height of ≈8 mm.

Phase purities were examined by XRD (Cu‐Kα, Rigaku 2500, Japan). High‐temperature XRD measurements (STOE STADI MP, Cu‐K_α1_ radiation, scan step of 0.015°) were performed up to 300 °C on a sealed glass capillary filled with a powdered sample diluted with small amount of quartz powder. The XRD data were collected at every 100 °C, and each lasted for 2 h. The existence and distribution of SiC particles were investigated by EPMA (JXA‐8230, JEOL, Japan). Microstructures of the bulk samples were observed by field emission scanning electron microscopy (JSM‐7001, JEOL, Japan). Diffuse reflectance measurements of finely ground powders were collected at RT using a Nicolet 6700 FT‐IR spectrometer. The band gap was calculated by the Dolgonos model.^47^ Electrical resistivity and Seebeck coefficient measurement at Tsinghua University were performed simultaneously on a ZEM‐2 apparatus (ULVAC‐RIKO, Japan) from 323 to 573 K in a helium atmosphere. Hall coefficient (*R*_H_) measurement at Tsinghua University was conducted using the Hall measurement system (ResiTest 8340DC, Toyo, Japan) from 323 to 573 K. Seebeck coefficient measurements at Northwestern University were carried out under high vacuum with a home‐built instrument by using Chromel–Nb thermocouples.^50^ Hall coefficients and resistivity (Van der Pauw, 4‐point probe) were also measured simultaneously at Northwestern University using a 2 T magnet with pressure‐assisted Molybdenum contacts.^51^ Hall carrier concentration (*n*_H_) and mobility (*µ*_H_) were then calculated by *n*_H_ = 1/(*eR*_H_) and *µ*_H_ = *R*_H_σ, where σ was electrical conductivity. The thermal diffusivity (*D*) was measured by the laser flash method (TC‐9000, Ulvac‐Riko, Japan and Netzsch LFA 457, Germany) at temperatures from 300 to 573 K. Finally, the thermal conductivity (κ) was calculated according to κ = *D* × *C*_p_ × *d*, in which *d* was the density of the material measured geometrically, and *C*_p_ was the specific heat measured by Simultaneous Thermal Analysis (STA 449F3, Netzsch, Germany) with a reference to the reported values.^35^ All the electrical and thermal transport properties were measured along the same direction perpendicular to the sintering pressing direction to avoid overestimating *zT* values. The transport properties measured at Tsinghua University and Northwestern University showed very close values. The uncertainty for *zT* values was considered to reach as high as 15–20%.^52^

## Conflict of Interest

The authors declare no conflict of interest.

## Supporting information

Supplementary
