# Supplementary material for: Self‐Tuning n‐Type Bi2(Te,Se)3/SiC Thermoelectric Nanocomposites to Realize High Performances up to 300 °C
Source: Adv Sci (Weinh). 2017 Aug 11;4(11):1700259. doi: 10.1002/advs.201700259 (PMC5700642; doi:10.1002/advs.201700259)
Supplement: Supplementary file 1 — Supplementary [file ADVS-4-na-s001.pdf]

Copyright WILEY-VCH Verlag GmbH & Co. KGaA, 69469 Weinheim, Germany, 2017.

ADVANCED SCIENCE Open Access

Supporting Information

for *Adv. Sci.,* DOI: 10.1002/advs.201700259

Self-Tuning n-Type $Bi_2(Te,Se)_3$/SiC Thermoelectric Nanocomposites to Realize High Performances up to 300 °C

Yu Pan, Umut Aydemir, Fu-Hua Sun, Chao-Feng Wu, Thomas C. Chasapis, G. Jeffrey Snyder, and Jing-Feng Li**

Copyright WILEY-VCH Verlag GmbH & Co. KGaA, 69469 Weinheim, Germany, 2013.

Supporting Information

Self-tuning n-type $Bi_2(Te,Se)_3$/SiC thermoelectric nanocomposites to realize high performances up to 300 °C

Yu Pan,[a,b] Umut Aydemir,[b] Fu-Hua Sun,[a] Chao-Feng Wu,[a] Thomas C. Chasapis,[b] G. Jeffrey Snyder,[b] and Jing-Feng Li*[a]*

Figures

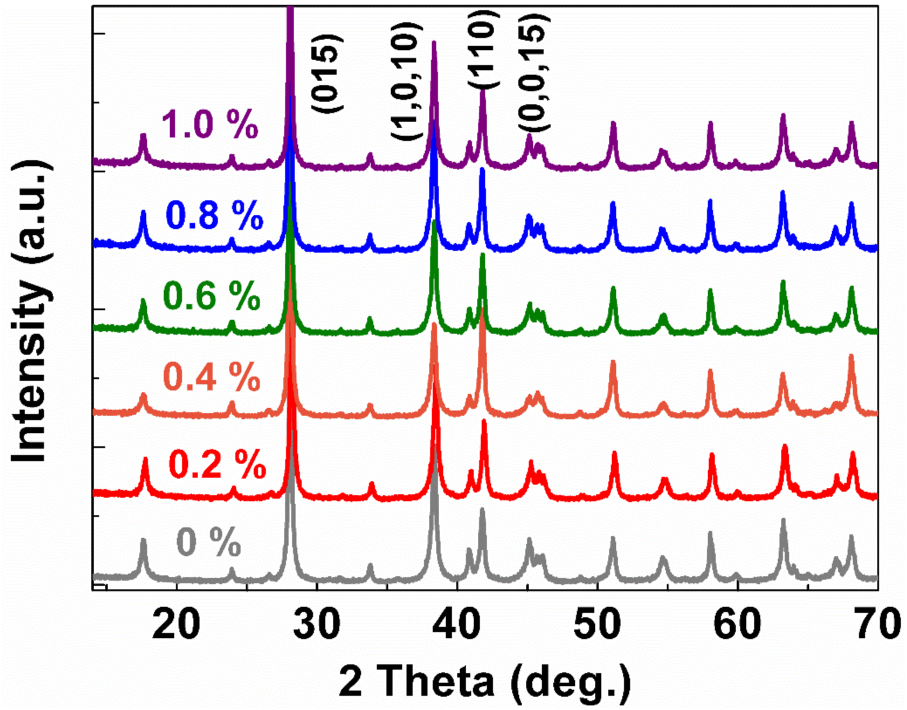

Figure S1. XRD patterns of $Bi_2Te_{2.2}Se_{0.8} - x$ vol.% SiC bulk samples (x = 0, 0.2, 0.4, 0.6. 0.8 and 1.0).

Figure S1 presents the XRD patterns of the $Bi_2Te_{2.2}Se_{0.8} - x$ vol.% SiC bulk samples (x = 0, 0.2, 0.4, 0.6. 0.8 and 1.0). None of the diffraction peaks of SiC is detected in the samples due to the low contents. Nevertheless, the existence and distribution of SiC is further confirmed by Electronic probe micro-analyzer (EPMA).

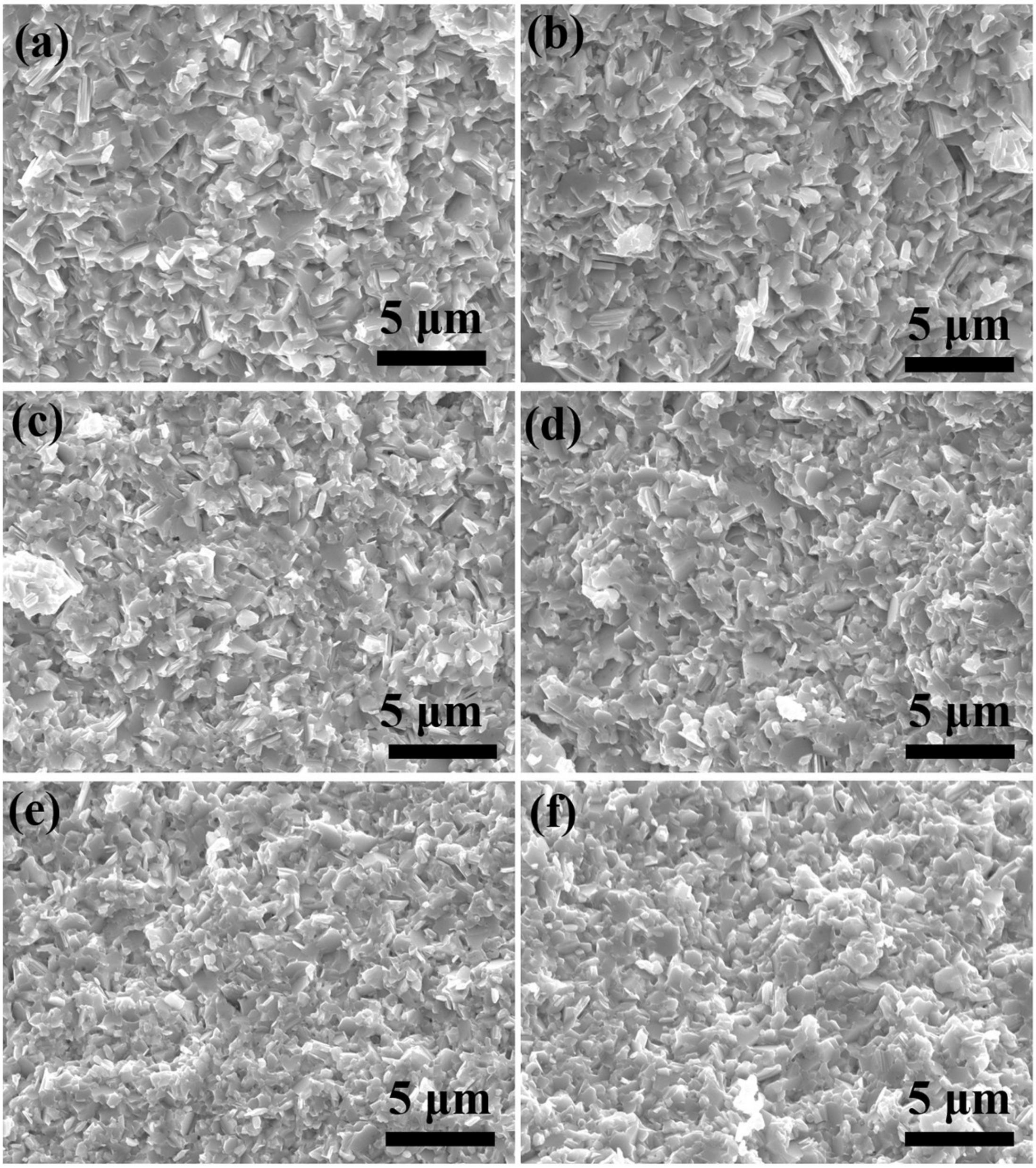

Figure S2. a-f) SEM images of the fractured surfaces of the $Bi_2Te_{2.2}Se_{0.8}$ – x vol.% SiC samples (x = 0, 0.2, 0.4, 0.6. 0.8, 1.0).

As shown in the SEM images in **Figure S2**, the samples display a relatively isotropic microstructure with fine grain structures despite the layered structure of $Bi_2Te_{3-x}Te_x$. Moreover, for the samples with higher contents of SiC, the grain domains become finer, which is also clearly illustrated by the gain domain size distribution, as shown in **Figure S3**. Therefore, it is considered that the SiC particles may decrease the grain domain size of the matrix.

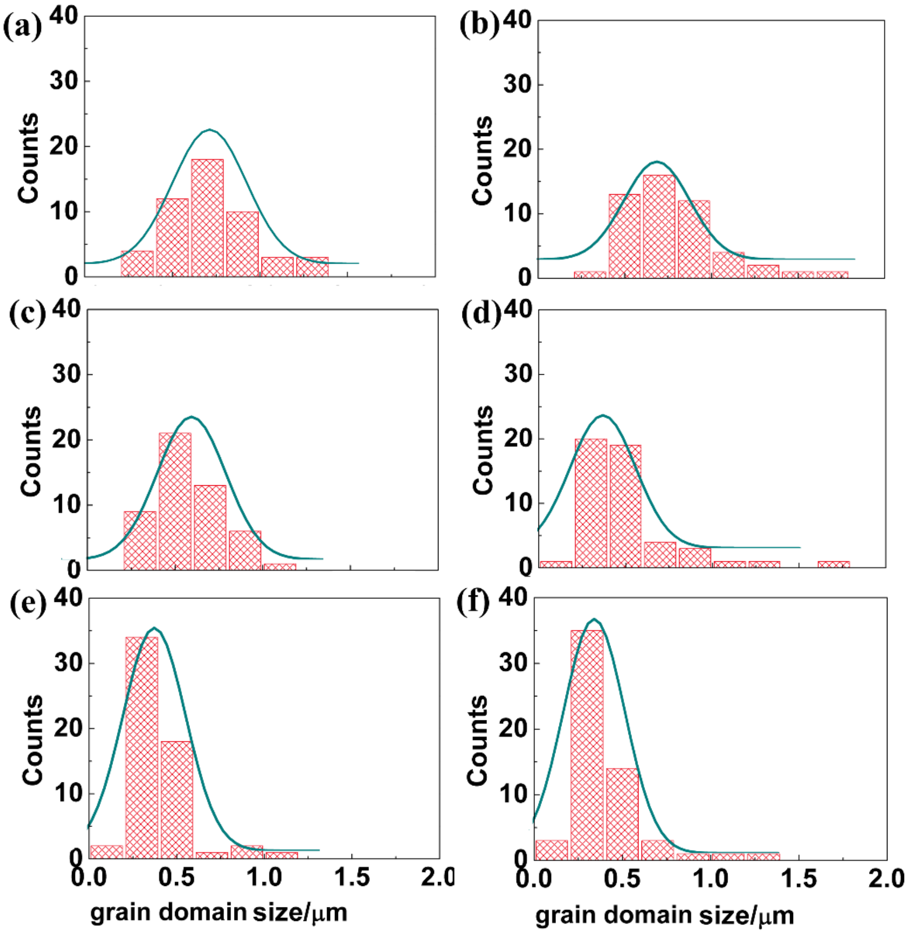

Figure S3. a-f) Grain domain size distribution of the $Bi_2Te_{2.2}Se_{0.8}$ – *x* vol.% SiC samples (*x* = 0, 0.2, 0.4, 0.6. 0.8, 1.0).

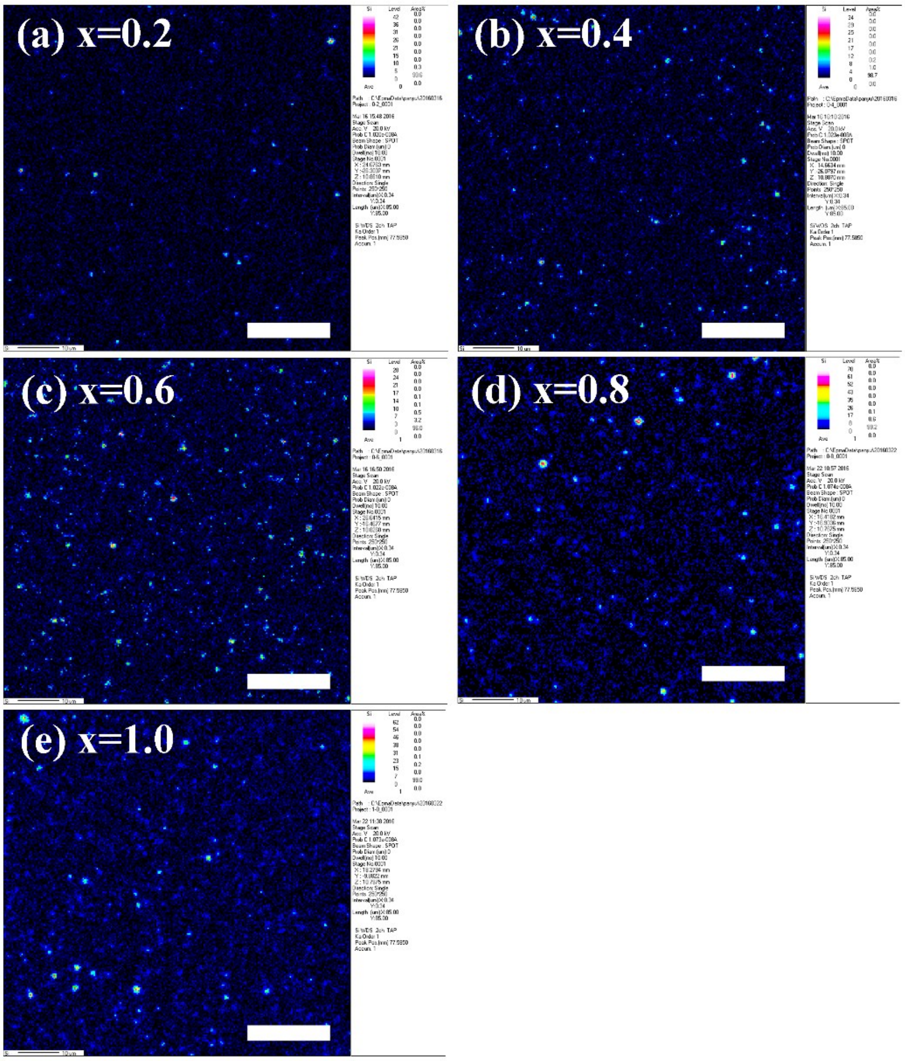

Figure S4. EPMA mapping of Si element on the polished surfaces of all the samples with SiC particles.

Figure S4 shows the EPMA mapping of Si element of all the samples with different amounts of SiC nanoparticles. It is clear that all the samples display Si-rich areas and SiC particles are distributed in the microstructure. Besides, more and more Si-rich areas are detected with increasing x, indicating that the practical amounts of SiC particles increases with the increasing nominal x.

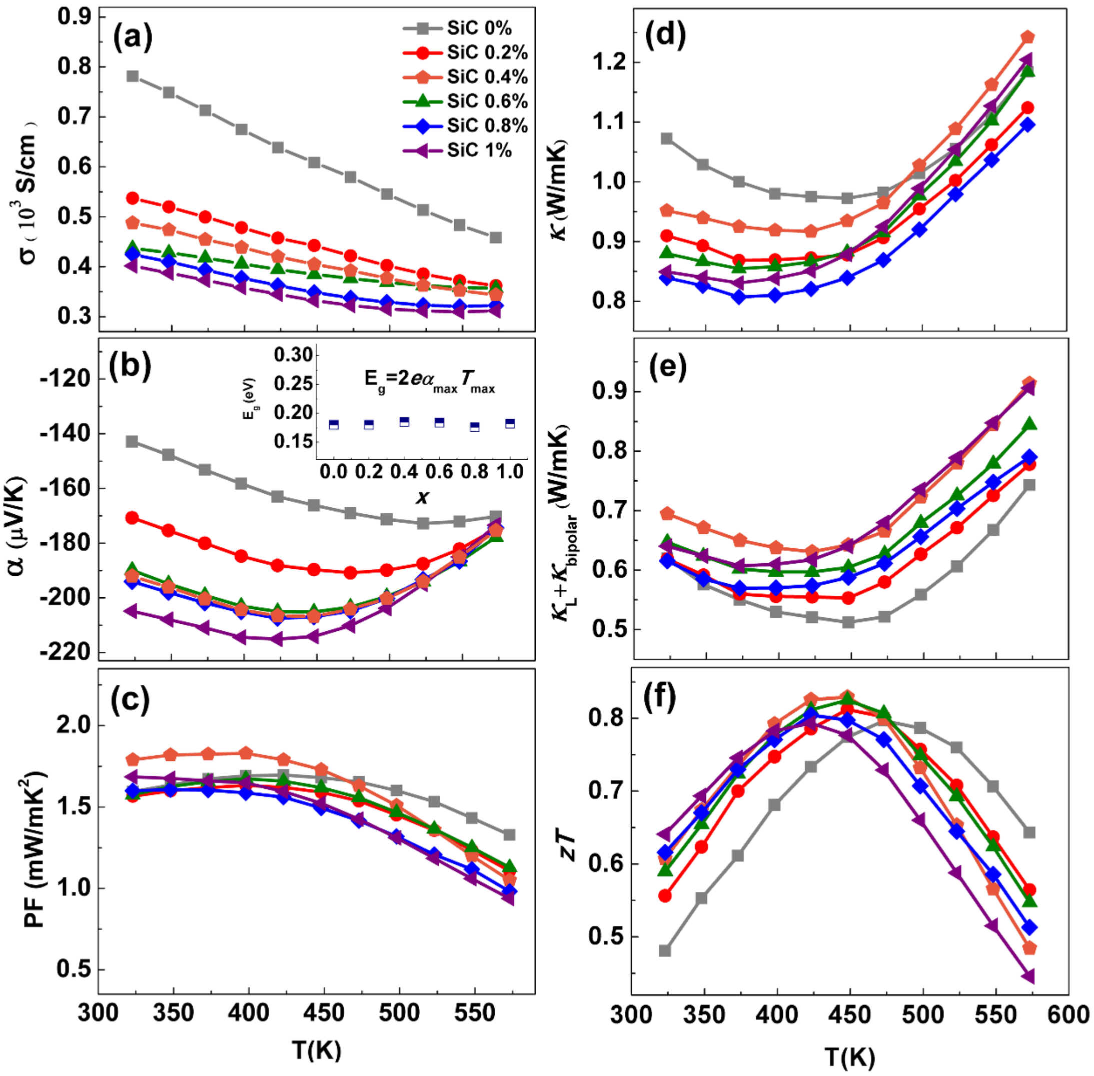

Figure S5. Temperature dependence of electrical and thermal transport properties of $Bi_2Te_{2.2}Se_{0.8}$ – x vol.% SiC: a) electrical conductivity, b) Seebeck coefficient (with an inset of energy gap), c) power factor, d) total thermal conductivity, e) lattice and bipolar thermal conductivity, and f) zT values.

The electrical transport properties of the $Bi_2Te_{2.2}Se_{0.8}$ – x vol.% SiC samples are investigated as a function of temperature. As shown in **Figure S5**a, the electrical conductivity displays negative temperature dependence, which is often found in degenerate thermoelectric semiconductors. Besides, the electrical conductivity decreases with SiC addition. Usually a decrease of electrical conductivity results in an improvement of Seebeck coefficient due to the opposite relation to carrier concentration. As shown in **Figure S5**b, the absolute values of Seebeck coefficient are indeed largely increased by SiC addition. On the other hand, the temperatures (T_{max}) that correspond to the maximum values of Seebeck coefficient shift to low temperatures with increasing SiC amounts. It is generally recognized that the T_{max} stands for the onset of intrinsic excitation and the energy gap could be obtained according to $E_g=2e\alpha_{max}T_{max}$.[1] The calculated results show that the band gap is rarely changed, as displayed in the inset of **Figure S5**b. **Figure S5**c show the values of power factor of all the samples. It is found that the power factors are nearly unchanged at low temperatures, except for the SiC-0.4 vol.% sample which shows slightly higher values. However, the power factors at higher temperatures of all the $Bi_2Te_{2.2}Se_{0.8}$-SiC composites are lower than the SiC-0 vol.% sample, which is because of the enhanced intrinsic excitation of minority carriers.

As shown in **Figure S5**d, the total thermal conductivities at low temperatures decreased for all the $Bi_2Te_{2.2}Se_{0.8}$-SiC composites, as compared to the SiC-0 vol.% sample. While the lattice thermal conductivity increases for $Bi_2Te_{2.2}Se_{0.8}$-SiC samples due to the high intrinsic thermal conductivity of SiC particles, as depicted in **Figure S5**e. This also indicates that the SiC dispersion has a weak or maybe ignorable effect on the scattering of phonons mainly since SiC particle is larger than the mean free path of the phonons. Additionally, the bipolar conduction due to intrinsic excitation of minority carriers also happens at lower temperatures with SiC contents, which is consistent with the Seebeck coefficients and carrier concentration. As a result, all the samples with SiC dispersion has higher *zT* values below 473 K, as shown in **Figure S5**f. However, the *zT* values rapidly decreases above 450 K, due to the promoted intrinsic excitation that dramatically increase the $\kappa_{bipolar}$ and largely decrease the Seebeck coefficient.

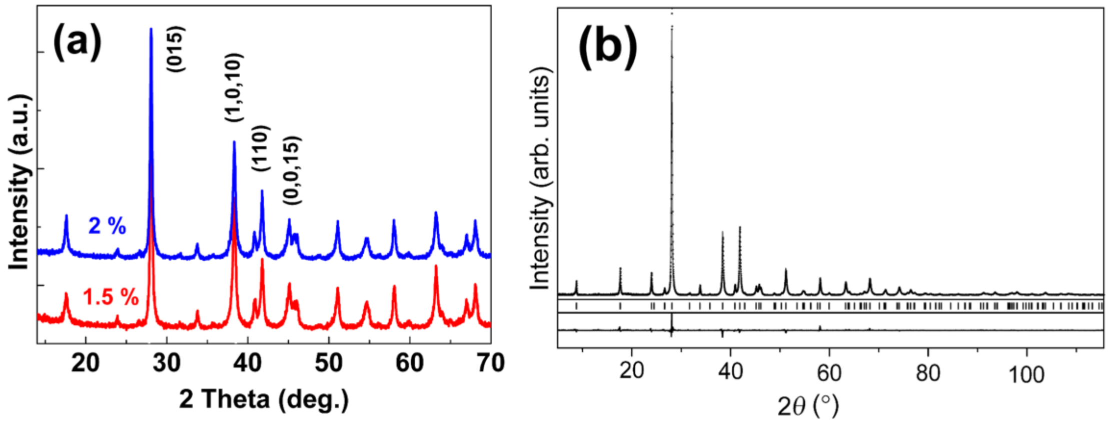

Figure S6. a) XRD results of the CuI doped bulk samples, b) Rietveld refinement plot for CuI-1.5 mol.% sample (Cu$K_{\alpha 1}$). Ticks mark the calculated reflection positions of the target phase and the baselines correspond to the residuals of the Rietveld refinement.

Figure S6a presents the XRD patterns of the CuI doped bulk samples. All the samples show a single phase of $Bi_2Te_{3-x}Se_x$ with no impurities detected. None of the diffraction peaks of SiC or CuI is detected in the samples due to the low contents. **Figure S6**b shows the Rietveld plot, where no pronounced texturing was observed, indicating an isotropic transport behavior of the as-prepared samples.

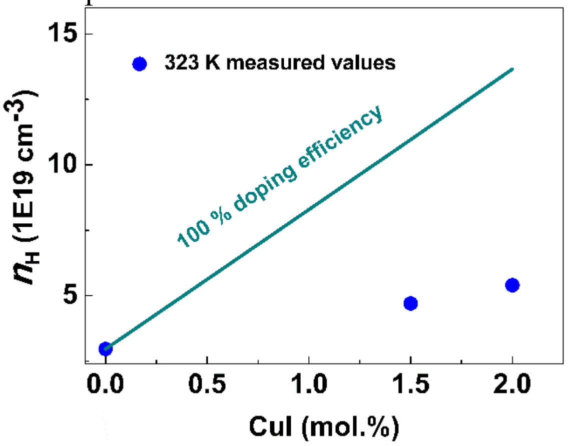

Figure S7. Measured Hall carrier concentration at 323 K as a function of nominal concentration of dopants, with comparison to the 100% doping efficiency of I.

Figure S7 displays the measured Hall carrier concentration ar 323 K as a function of nominal concentration of dopants. Here the 100% doping efficiency is calculated based on the replacement of I^- at Te^{2-}/Se^{2-} sites, which donated one electron per I^-. It can be seen that the carrier concentration is very far away from the 100% doping efficiency, indicating that the solubility of Cu/I in the lattice is relatively low.

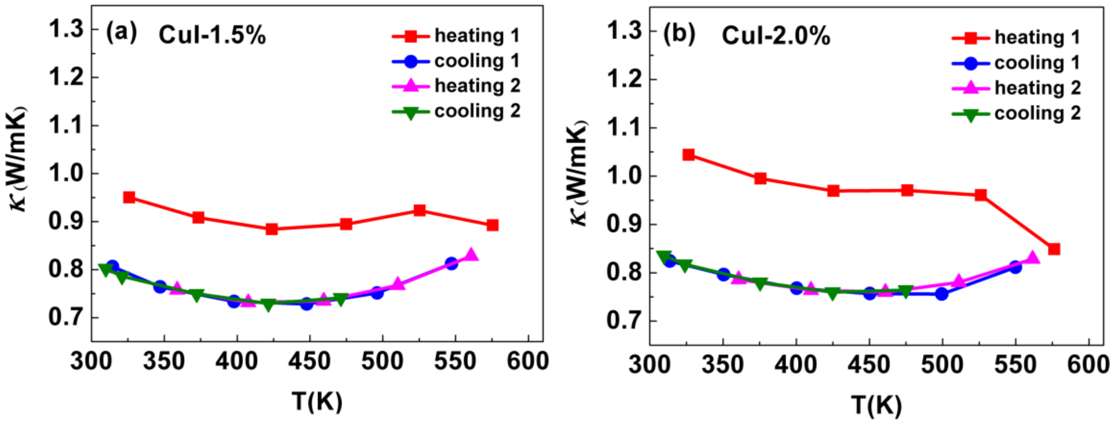

Figure S8. Temperature dependent thermal condcutivity of the doped samples for two cycles: a) CuI-1.5 mol.% and b) CuI-2 mol.%.

Figure S8 shows the temperature dependent thermal conductivity of the doped samples for two cycles. It could be seen that the thermal conductivity remains stable after the first heating. All the transport properties should be stable as the representative thermal conductivity.

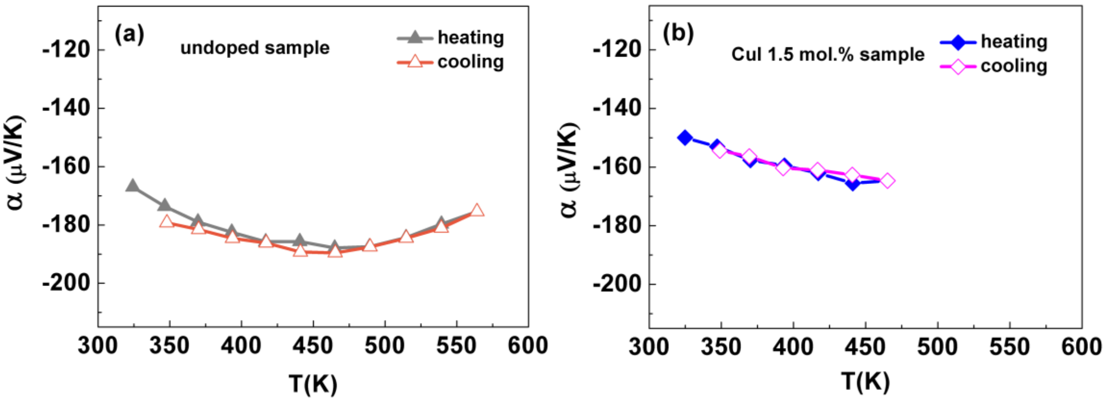

Figure S9. Temperature dependent Seebeck coefficient of a) undoped sample from 323 K to 573 K, and b) CuI-1.5 mol.% sample from 323 K to 473 K for heating and cooling.

Figure S9a shows the temperature dependent Seebeck coefficient of the undoped sample from 323 K to 573 K for both heating and cooling. Here the Seebeck coefficient measurement is used as an example to show that the undoped sample displays no hysteresis between heating and cooling, indicating that the differences between before and after heating are not caused by the fabrication method but may related to the Cu/I dopants. **Figure S9**b show the temperature dependent Seebeck coefficient of the CuI-1.5 mol.% sample from 323 K to 473 K for both heating and cooling. It is demonstrated that there is no hysteresis between two subsequent measurements if the measurement temperature is limited to 200 °C.

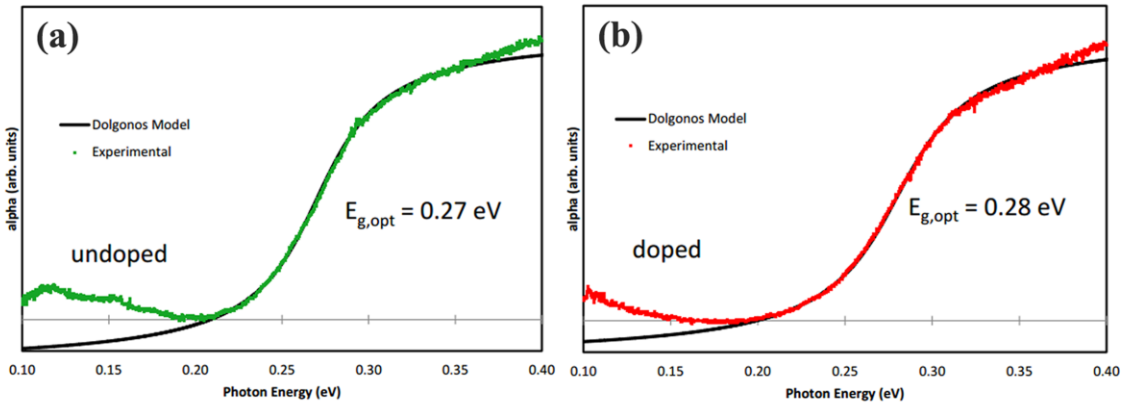

Figure S10. Optical band gap by diffuse reflectance analysis in a) undoped and b) Cu/I doped $Bi_2Te_{2.2}Se_{0.8}$ – 0.6 vol.% SiC composites.

The undoped and doped samples show very close optical band gap values, indicating that Cu/I doping hardly change the band gap. The band gap in this system is calculated by Dolgonos model[2] which is more appropriate for degenerate semiconductors than extrapolating the linear region of the absorption curve to the *x*-axis.

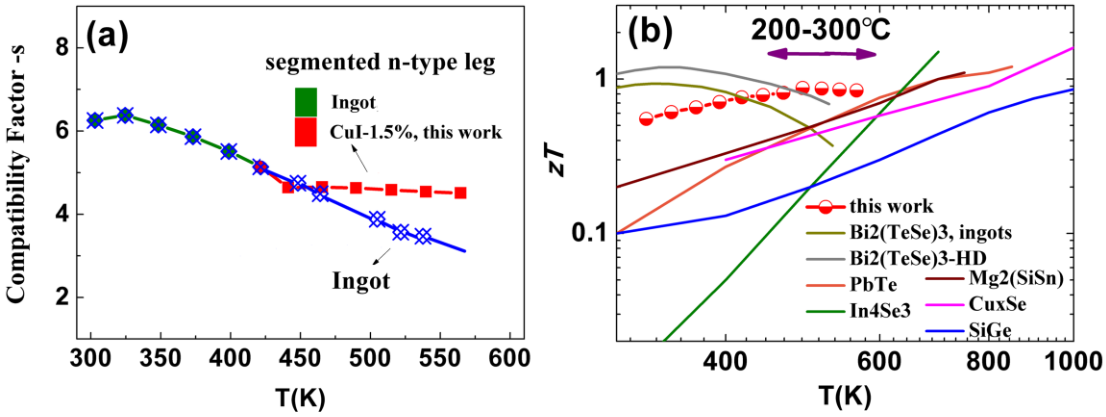

Figure S11. a) Material *zT* comparison to different both low and high performance n-type thermoelectric materials ($Bi_2(TeSe)_3$ ingots[3], $Bi_2(TeSe)_3$ hot deformed polycrystalline[3], PbTe[4], In_4Se_3[5], $Mg_2(SiSn)$ [6], Cu_xSe[7] and SiGe[8]), and b) thermoelectric compatibility factor (-*s*) of n-type $Bi_2Te_{2.79}Se_{0.21}$ ingots[3] and that of a segmented leg composed of ingots[3] for 300-423 K and CuI-1.5% doped $Bi_2Te_{2.2}Se_{0.8}$ (this work) for 423-573 K.

The greatly enhanced *zT* values at 200 - 300 °C is of significance as few materials show high *zT* in this temperature range, as shown in **Figure S11**a. For a segmented design, small change of compatibility factors of the two materials are desired. As shown in **Figure S11**b, the compatibility factors (-*s*) vary little with temperature (6.3-4.5) along the segmented leg, in which the variation is even smaller than the non-segmented ingots leg (6.3-3.3).

Tables

Table S1 Comparison of lattice parameters for samples with and without SiC.

Lattice parameters	$Bi_2Te_{2.2}Se_{0.8}$	$Bi_2Te_{2.2}Se_{0.8}$-0.6 vol.% SiC	$Bi_2Te_{2.2}Se_{0.8}$-0.6 vol.% SiC-1.5 mol.% CuI
**a (Å)**	4.3171(1)	4.3158(1)	4.3173(1)
**c (Å)**	30.0856 (6)	30.0848(6)	30.0971(6)

Table S2 Atomic coordinates, displacement parameters (U_{iso}, in Å^2) and site occupancy factor (*SOF*) of $Bi_2Te_{2.2}Se_{0.8}$ with 1.5 mol.% CuI added (space group of *R*-3*m*; R_i = 0.04; R_p = 0.08). Since the amounts of Cu and I are extremely small in this sample, their occupancy contributions are omitted. Standard deviations are provided in parentheses.

Atom	Site	*x*	*y*	*z*	U_{iso}	*SOF*
Bi	6*c*	0	0	0.39688(3)	0.0191(3)	1
Te(1)	6*c*	0	0	0.78852(4)	0.0172(5)	1
Te(2)	3*a*	0	0	0	0.0193(9)	0.618(8)
Se	3*a*	0	0	0	0.0193(9)	0.382(8)

References

[1] D. M. Rowe, *Materials, preparation, and charaterization in thermoelectrics* **2012**, CRC Press, Boca Raton London, New York.
[2] A. Dolgonos, T. O. Mason, K. R. Poeppelmeier, *J. Solid State Chem.* **2016**, 240, 43-48.
[3] L. Hu, H. Wu, T. Zhu, C. Fu, J. He, P. Ying, X. Zhao, *Adv. Energy Mater.* **2015**, 5, 1500411.
[4] Y. Pei, Z. M. Gibbs, A. Gloskovskii, B. Balke, W. G. Zeier, G. J. Snyder, Optimum carrier concentration in n-type PbTe thermoelectrics. *Adv. Energy Mater.* **2014**, 4, 1400486.
[5] J. S. Rhyee, K. H. Lee, S. M. Lee, E. Cho, S. I. Kim, E. Lee, Y. S. Kwon, J. H. Shim, G. Kotliar, *Nature*, **2009**, 459, 965-968.
[6] G. Jiang, J. He, T. Zhu, C. Fu, X. Liu, L. Hu, X. Zhao, *Adv. Funct. Mater.* **2014**, 24, 3776-3781.
[7] H. Liu, X. Shi, F. Xu, L. Zhang, W. Zhang, L. Chen, Q. Li, C. Uher, T. Day, G. J. Snyder, *Nature Mater.* **2012**, 11, 422-425.
[8] J. P. Dismukes, L. Ekstrom, E. F.Steigmeier, I. Kudman, D. S. Beers, *J. Appl. Phys.* **1964**, 35, 2899–2907.
